# Accuracy of Base of Support Using an Inertial Sensor Based Motion Capture System

**DOI:** 10.3390/s17092091

**Published:** 2017-09-12

**Authors:** Liangjie Guo, Shuping Xiong

**Affiliations:** 1 Department of Safety Engineering, China University of Geosciences, Wuhan 430074, China; guoliangjie@yahoo.com; 2 Human Factors and Ergonomics Laboratory, Department of Industrial & Systems Engineering, Korea Advanced Institute of Science and Technology (KAIST), Daejeon 34141, Korea

**Keywords:** inertial sensor, motion capture, Xsens MVN, accuracy, base of support, biomechanical model

## Abstract

The potential of miniature inertial sensors for human balance and gait analysis appears promising. Base of support (BOS), together with its interaction with center of mass, is a critical indicator in above mentioned research fields. This study aims to evaluate the accuracy of Xsens MVN BIOMECH, a commercial widely used inertial sensor-based motion capture system, for measuring static BOS and examine the effect of different task complexity on the accuracy. Eleven young males participated in this study and went through eleven different experimental tasks. Results showed there were considerable errors in estimating BOS area (error ranged from −12.6% to +64.6%) from Xsens MVN and a large error in foot separation distance when there was knee flexion. The estimated BOS area from MVN was smaller than the ground truth from footprint when there was no knee flexion, and larger when there was knee flexion, and it increased monotonically along with the knee flexion angles. Wrongly estimated foot separations, mainly caused by knee flexion, and the initial system estimation error on BOS, were two major reasons for error and instability of BOS estimation. The findings suggested that caution should be taken when using Xsens MVN BIOMECH to estimate BOS and foot position-related measurements, especially for postures/motions with knee flexion.

## 1. Introduction

Miniature inertial sensors are cost effective, wearable, compact and lightweight, and have been widely used in many fields. Especially, inertial sensor technology makes it possible to capture human motion and acquire kinematics in an outdoor environment, without the drawbacks of optical motion capture systems, such as restriction to a laboratory environment and markers occlusion [1]. Due to the rapid development of inertial sensing technology and human motion reconstruction techniques, it has become possible to use a few inertial sensors for reconstructing appealing human motions in different scenarios [2,3]. The Xsens MVN BIOMECH (Xsens Technologies B.V., Enschede, The Netherlands) is a commercially available inertial sensor-based motion capture system composed of 17 miniature inertial sensors placed over the full body [4,5]. It has been widely used in the research fields of biomechanics [6,7,8,9], ergonomics and human factors [10,11], sports science [12,13,14,15,16,17,18] and virtual or augmented reality [19,20,21]. Xsens MVN has a good accuracy in human kinematics estimation, such as joint angle and segment orientation [22,23,24,25,26], it has been validated against optical motion capture system [7,26,27] and is currently considered as the ‘gold reference’ for kinematics measurements [28,29].

Recently, the Xsens MVN is increasingly being used in the assessment of human body balance or postural stability [30,31,32,33,34]. Maintaining body balance is essential for humans to perform the daily activities, and people with postural instability would be confronted with difficulties in completing desired actions [35,36]. The condition for human stability, which was already well-known, is the confinement of the center of mass (COM) in static situations or extrapolated center of mass (XCOM) in dynamic situations within the base of support (BOS) [37,38,39]. The COM–BOS interaction is indicative of both static and dynamic balance control ability [40,41], and is more sensitive in distinguishing deviations in balance control in elderly people [40]. The knowledge of BOS and COM trajectory could help in rehabilitation for patients who has balance disorders [42]. Besides that, Xsens MVN has been widely used in gait analysis [6,7,8,9,32], in which margin of stability (MOS, determined by COM or XCOM position relative to BOS boundaries), step width and length (related to side length of BOS) are important outcome measures [40,41,43,44,45,46,47] of gait stability. Actually, MOS reflects the COM-BOS interaction, and is influenced by voluntary changes in two gait parameters (step width and length) and can be increased by longer steps (larger anterior-posterior BOS) and wider steps (larger medial-lateral BOS) [41,48,49]. In addition, BOS-related gait parameters, step width and length, themselves, were also important measures for gait analysis [31,50].

The latest version of Xsens MVN can provide the COM position of whole body and landmarks positions of each foot in MVN Studio, which makes it possible to obtain COM and BOS simultaneously for assessing human balance and gait stability. Many studies have been conducted on validating Xsens MVN for COM position and related applications. Dinu et al. [51] investigated the accuracy and reliability of Xsens MVN in whole body COM position estimation. Using Vicon Motion systems (Vicon Motion Systems Ltd., Oxford, UK) as the reference, they reported small mean differences (5.45 mm, 3.25 mm and 0.73 mm on X, Y and Z component separately) between the two systems. Koenemann et al. [52] extracted relative COM position data (COM of whole body relative to COM of both feet) from MVN Studio to drive a robot real time, in which the robot imitated well with a high stability and similarity to real human motions. Michnik et al. [30] used Xsens MVN to measure COM position and its speed to study the similarities and differences of body control during professional, externally forced fall to the side performed by men aged 24 and 65 years. Damian et al. [21] used the position and orientation data provided by MVN Studio to synchronize the real and virtual environments and to create intuitive interaction modalities with virtual entities in the augmented reality environment. Carson et al. [13,15,53] used the Xsens MVN position data as the input of Visual3D software (C-Motion Inc., Germantown, MD, USA) to analyze movement variability and identify swing events of male golfers. Doric et al. [19] used Xsens MVN and Oculus Rift Development Kit 2 (Oculus VR LLC, Irvine, CA, USA) to develop a pedestrian simulator to study the crossing behavior and risk acceptance, in which position data from Xsens biomechanical model was used as one kind of inputs.

Even though the majority of aforementioned studies showed that Xsens MVN had reasonably good performance in estimating the position of whole body COM, there was no information on the performance of Xsens MVN system in BOS measurements. This limits the potential of applying Xsens MVN system into human balance and gait analysis due to the lack of knowledge on COM–BOS interaction. Therefore, the main objective of this study is to evaluate the accuracy of BOS obtained from Xsens MVN system. In this study, we focus on static BOS (both feet remain still) with different body postures/motions due to the following reasons: (1) static BOS is easy to obtain and measure without complex experimental setup; (2) static BOS at different body postures is an important variable used by many studies for assessing human balance and postural stability [54,55,56]; (3) having good accuracy on static BOS should be a necessary condition for acceptable accuracy on dynamic BOS for Xsens MVN system, therefore, the first critical step in assessing accuracy of Xsens-based BOS is to examine the accuracy of static BOS using the Xsens MVN system.

## 2. Methodology

### 2.1. Participants

Eleven healthy young males (age 26.1 ± 4.2 years; height 171.0 ± 6.1 cm; weight 72.4 ± 8.1 kg) without any restriction on physical activity and having no self-reported recent history of musculoskeletal issues participated in this study. All subjects were recruited from a convenience sample of postgraduate students at Korea Advanced Institute of Science and Technology (KAIST, Daejeon, Korea). Each subject signed an informed consent approved by KAIST Institutional Review Board (IRB) before participating in the experiment.

### 2.2. Experimental Design and Procedure

A within-subject repeated measures trial was utilized. Each participant underwent eleven different postural tasks and one N-pose calibration task. Different static postures and dynamic motions (Table 1) were designed in this experimental study to investigate the effect of postures/motions on the accuracy of Xsens-based BOS.

(1) Experimental apparatus

**Figure 1:**
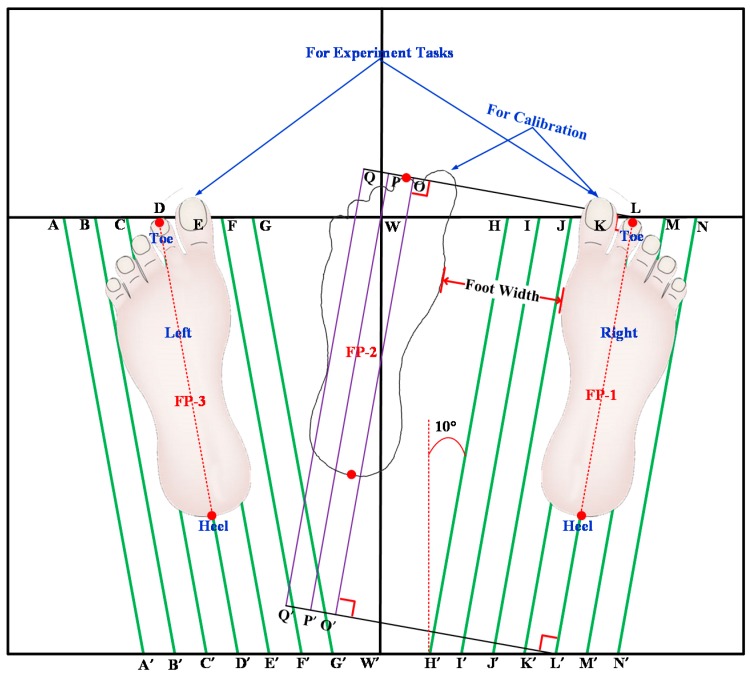
Paper for footprint acquisition and related foot positions.

An Xsens MVN BIOMECH motion capture system powered by its matching software Xsens MVN Studio (version 4.3.0) was used to record the participants’ kinematics data, including feet position data, at the frequency of 240 HZ. One large size paper with pre-drawn reference lines (see the green and purple lines in Figure 1) posted on a wooden board, was used to acquire each participant’s footprints (both left and right feet), serving as the golden reference of BOS. Non-slip socks were provided to help participants keep their feet still during experimental tasks. Marking pens, surgical tapes were used to mark the exact positions of 2nd toes, 1st and 5th metatarsophalangeal (MTP) joints and heel centers.

(2) Experimental tasks

Each participant was asked to perform eleven different experimental tasks (T1–T11) and one calibration task T0 (Table 1). T0–T3 were static postures (Section 1, S1) and the other eight tasks were dynamic motions (S2–S4). T4–T6 (S2) were upper body motions, T7–T9 (S3) were lower body motions (without knee flexion) that the upper body was kept still relative to the lower body, and T10–T11 (S4) were knee flexion/extension motions. There was no any movement of both feet in all the tasks. Each participant was asked to keep each static task (S1) for 20 s and to perform each dynamic task (S2–S4) in normal speed for eight loops continuously in each trial, three trials in total for each task. Schematic representations of each experimental task are shown in Appendix A. Unless otherwise noted in the following, calibration task was T0, experimental tasks were T1–T11.

(3) Experimental procedure

**Figure 2:**
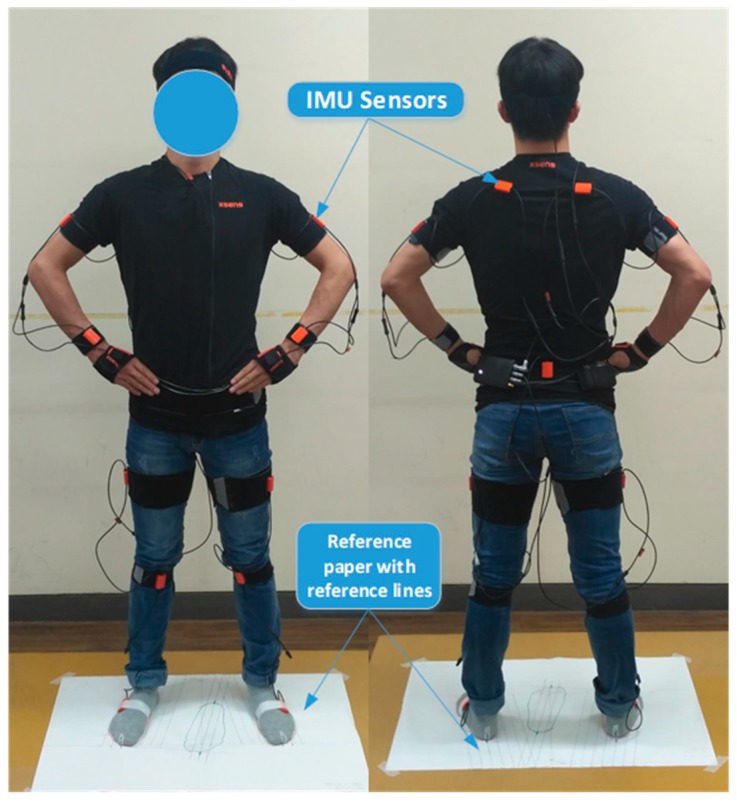
Experimental setting with inertial sensor based Xsens MVN BIOMECH full body suit and footprint papers.

The experiment was conducted in a quiet, temperature controlled room. The total experimental time of each participant was around 80 min. The brief details of the testing procedure were as follows:

(a) Manually measure subject anthropometric dimensions: body weight, body height, foot length, foot width, shoulder width and the distance between 1st and 5th MTP joints.
(b) Determine foot positions on a large size paper and then acquire footprints in calibration and experimental tasks (Figure 1). For the calibration position (FP-1 and FP-2 in Figure 1 for T0), the right foot was at FP-1, and the left foot was at FP-2, which was determined by strictly following the calibration instructions from Xsens. For the experimental positions (FP-1 & FP-3 in Figure 1 for T1–T11), the width between feet was determined by taking into account the participant’s shoulder width and self-reported comfort level, and toe-out angle was kept to be 10 degrees for easiness when performing all experimental tasks, especially squatting-related tasks. The footprints were directly drawn on the paper after foot positions were determined, and the positions of 2nd toes and heel centers were marked on the footprint paper.
(c) Ask the subject to wear the Xsens MVN BIOMECH system by using velcro straps and a Lycra T-shirt provided by Xsens (see Figure 2). According to the instructions of the Xsens system, the 17 IMU sensors were attached on back of the head, pelvis, sternum, shoulders (right and left, R&L), upper arms (R&L), forearms (R&L), hands (R&L), upper legs (R&L), lower legs (R&L) and feet (R&L). Two kinds of calibrations were carried out afterwards in N-pose. One was the Segment Calibration (SC) that was required to align the motion trackers to the segments of the subject. The other one was an Additional Calibration (AC) for X-axis definition, in which the direction the subject was facing defined the new X-axis.
(d) Record experimental data at different tasks. Each participant performed different tasks in given order (Table 1) within one section, but in random order for four sections, meanwhile the kinematics data, including position data, was recorded by MVN Studio. To reduce accumulated error over time, both SC and AC were carried out before starting a new task, and AC was performed before starting a new trial. A practice session prior to testing was given to familiarize participants with the different postural and motion tasks.

### 2.3. Data Processing and Outcome Measures

(1) Data processing

**Figure 3:**
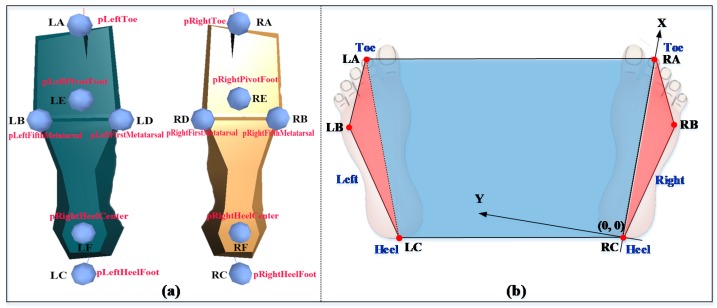
(**a**) Landmarks of foot soles in biomechanical model of MVN Studio; (**b**) Xsens-based BOS.

Direct manual measurements of each participant’s feet and footprints were considered as the reference or ground truth of BOS related measures. For Xsens data, MVN Studio collected and stored all the data in a proprietary file format (*.mvn), which was exported into two kinds of file formats (*.c3d and *.mvnx). Feet position data was extracted from c3d files by using Motion Kinematic & Kinetic Analyzer (Mokka, version 0.6) software (http://biomechanical-toolkit.github.io/mokka/ index.html). Figure 3a shows a screenshot of foot soles in the biomechanical human model created by MVN Studio, and there were six landmarks on the sole of each foot. For each landmark, 3D coordinates were acquired from c3d files. In this study, each foot, for example the left foot, only the position data on horizontal plane of the following four landmarks was used: pLeftToe (LA), pLeftFifthMetatarsal (LB), pLeftHeelFoot (LC) and pLeftFirstMetatarsal (LD). It is important to note that after the Additional Calibration for X-axis definition, the point of pRightHeelFoot (RC) was set as the origin (0, 0) of the coordinate system. Joint angle data was extracted from mvnx files by using the MATLAB code provided by Xsens, which was used to identify key feature points (e.g., the start and end timepoints) in each loop according to the troughs and peaks of the angle data in dynamic experimental tasks (T4–T11). For static tasks (T1–T3), the whole time frame of each task was equally divided into 8 segments to match with 8 loops in dynamic tasks for a possible cross comparison, aiming to check the changes of Xsens-based BOS at different experimental tasks.

(2) Outcome measures

The Quadrilateral LA-LC-RC-RA (see Figure 3b) was used to approximate BOS. The BOS was measured in terms of size (area and side length) and position (four vertexes and one central point) during the experimental tasks (Table 2). Relative Error (RE, %) in BOS size and absolute error in position (Position Error, PE, mm) were used to quantify the accuracy of Xsens-based BOS, which were calculated using Equations (1) and (2). The changes in size and position of Xsens-based BOS could reflect the effects on BOS of different tasks, which were measured by Average Range (ARG, cm^2^ or mm) and its percentage (PARG, %). ARG and PARG were derived using Equations (3) and (4):

(1) $$RE=\frac{XV\;-\;RV}{RV}\;\times 100\%$$

(2) $$PE=\sqrt{{\left(X_{X}-X_{R}\right)}^{2}+{\left(Y_{X}-Y_{R}\right)}^{2}}$$

where *XV* is the value of BOS size based on Xsens data, *RV* is its corresponding reference value; (*X*_X_, *Y*_X_) is coordinate of four vertexes (2nd toes and heels of both left and right feet) and central point based on Xsens data, (*X*_R_, *Y*_R_) is the corresponding reference coordinate;

(3) $$ARG=\frac{\sum_{i=1}^{m}\sum_{j=1}^{n}\left(Max_{ij}-Min_{ij}\right)\;}{m\times n}$$

(4) PARG=ARG/RV

where *m* and *n* are the number of trials and the number of loops, *Max_ij_* and *Min_ij_* are the maxima and minima values of the target measure in Loop *j* at Trial *i* from Xsens data (*XV*), *RV* is the reference value.

### 2.4. Statistical Analysis

All outcome measures were computed using MATLAB (v. R2016b, Math Works Inc., Natick, MA, USA) and only the data of first seven loops was used for each experimental task to minimize the impact of under or over-recording of Xsens data in the last loop. Statistical analysis, like Analysis of Variance (ANOVA), was processed by using SPSS (v. 24.0, IBM Corp., Armonk, NY, USA). In the ANOVAs, Boxplot was used to detect potential outliers, Shapiro-Wilk test was used for normality test, and Greenhouse-Gasser correction was used for the violation of the spherical assumption, which was assessed by Mauchly‘s test of sphericity.

## 3. Results

### 3.1. The Accuracy of Xsens-Based BOS

#### 3.1.1. Accuracy of BOS size

(1) Relative error in area

**Figure 4:**
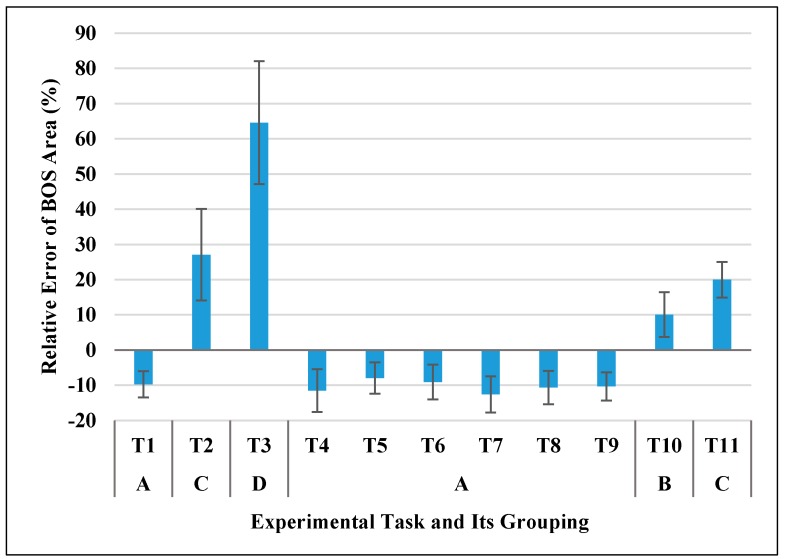
Relative errors of Xsens-based BOS area in different experimental tasks and post-hoc grouping result (Same letters indicate no significant difference between experimental tasks).

**Figure 5:**
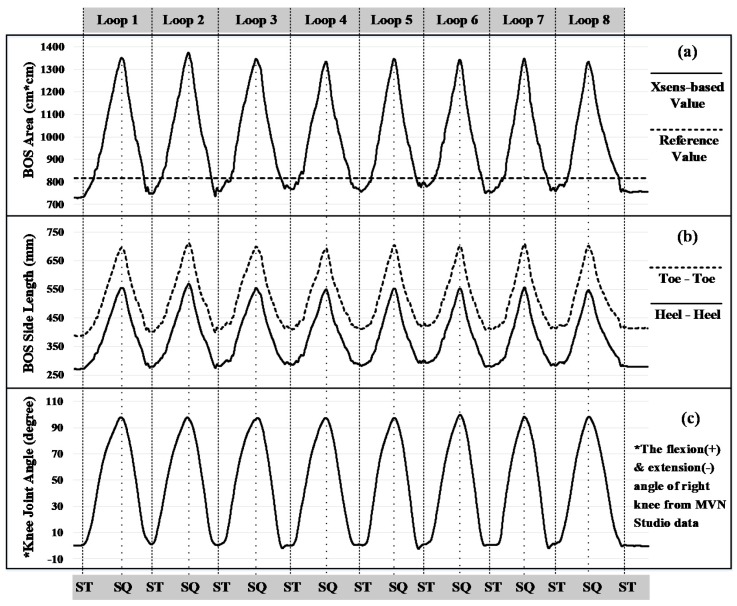
A typical example of the changes of (**a**) Xsens-based BOS area and (**b**) side lengths: Toe-Toe and Heel-Heel distances, along with (**c**) knee flexion/extension angle in a Standing-Full Squatting motion (Task 11). ST: Standing, SQ: Squatting.

Figure 4 shows the relative errors (RE) of the Xsens-based BOS area in different experimental tasks (T1-T11). The data in all tasks was normally distributed but the assumption of sphericity was violated, therefore a Greenhouse-Geisser correction was applied. ANOVA results showed the relative error of BOS area was significantly different in the different tasks (*F* (1.929, 19.286) = 152.552, *p* < 0.0005). Post-hoc comparisons showed that the eleven experiment tasks can be categorized into four groups (see A, B, C and D in Figure 4), tasks that without knee flexion/extension (T1, T4–T9) were in Group A; standing to half squatting motion (T10) was in Group B; static half squatting posture (T2) and standing to full squatting motion (T11) were in Group C; static full squatting posture (T3) was in Group D. A steady increase in the average RE of BOS area can be observed from Group A to D. Furthermore, when there was no knee flexion (Group A), the area of Xsens-based BOS was always smaller than the real (−10.3% in average). When there was knee flexion (Groups B, C and D), the area was always larger than the real (+30.4% in average) and increased significantly with a bigger knee flexion angle (see a typical example in Figure 5a,c). The accuracy in BOS area firstly increased (RE: from negative towards zero) and then decreased (RE: from zero towards positive) when performing standing to squatting motions (T10, T11).

(2) Relative error in side length

(a) Toe-toe distance and heel-heel distance (medial-lateral direction)

**Figure 6:**
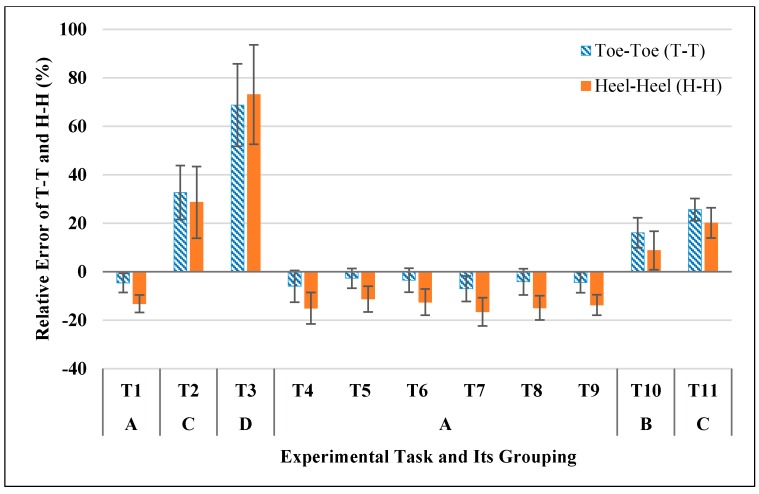
Relative errors of Xsens-based BOS side lengths (Toe-Toe, T-T and Heel-Heel, H-H) in different experimental tasks and post-hoc grouping result (Same letters indicate no significant difference between experimental tasks).

Figure 6 shows the relative errors (RE) of Xsens-based toe-toe (T-T) and heel-heel (H-H) distances. RE data of T-T and H-H was normally distributed but the assumption of sphericity was violated, therefore a Greenhouse-Geisser correction was applied. Both the RE of T-T and RE of H-H were significantly different in the different tasks (T-T: *F* (2.015, 20.153) = 170.886, *p* < 0.0005; H-H: *F* (1.974, 19.738) = 152.448, *p* < 0.0005). Post-hoc comparisons showed that the eleven experiment tasks could be categorized into four groups based on relative errors of side lengths (see A, B, C and D in Figure 6). The grouping results for T-T and H-H were exactly the same, which was also the same as that for RE of BOS area. In addition, when there was no knee flexion (Group A), two side lengths, T-T and H-H, were smaller than the real (−4.6% for T-T and -13.9% for H-H in average). When there was knee flexion (Groups B, C and D), both T-T and H-H were always larger than the real (+35.8% for T-T and +32.7% for H-H in average). When performing standing to squatting motions (T10, T11), the foot separation distances (toe-toe, heel-heel) increased significantly with a larger knee flexion angle (see Figure 5b,c).

(b) Feet length (anterior-posterior direction)

The accuracy of foot length was very high and the average relative error (RE) was −0.043% (range: −0.221%~−0.003%). Furthermore, there was negligible difference in RE of foot length in different tasks (all mean differences were within 0.064%).

#### 3.1.2. BOS Position Error

**Figure 7:**
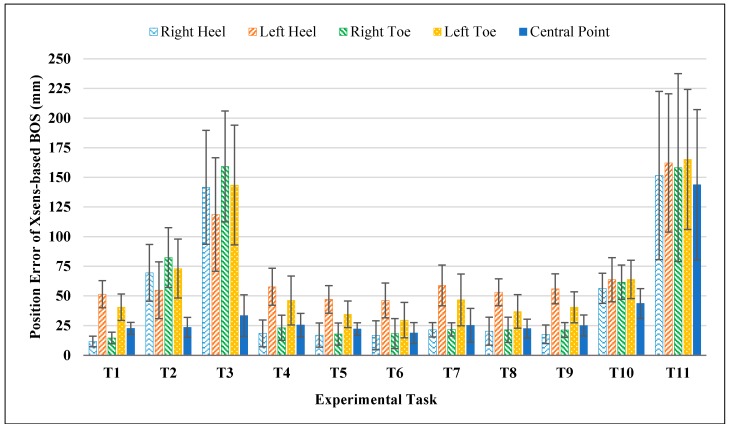
Position errors of Xsens-based BOS in different experimental tasks.

Figure 7 shows position errors (PE) of BOS. When there was no knee flexion (T1, T4–T9), the position error of right foot position (Right Toe: 19.8 mm, Right Heel: 17.6 mm) was smaller than the left foot (Left Toe: 39.3 mm, Left Heel: 52.9 mm). When there was knee flexion (T2–T3, T10–T11), no considerable differences on position errors were found between right (Right Toe: 115.3mm, Right Heel: 104.7 mm) and left (Left Toe: 111.4 mm, Left Heel: 99.8 mm) feet, even though the errors were much larger compared to no knee flexion tasks. In squatting-related tasks (T2, T3, T10 and T11) especially static squatting tasks (T2, T3), the position error of central point was smaller than four vertexes (toes and heels).

### 3.2. Change of Xsens-Based BOS in Different Experimental Tasks

According to the definition and formula of average range (ARG) and its percentage (PARG) in Equations (3) and (4), the larger value of PARG or ARG was, the bigger the change in BOS size/position during the task.

#### 3.2.1. Change of BOS Size

(1) Change in area

**Figure 8:**
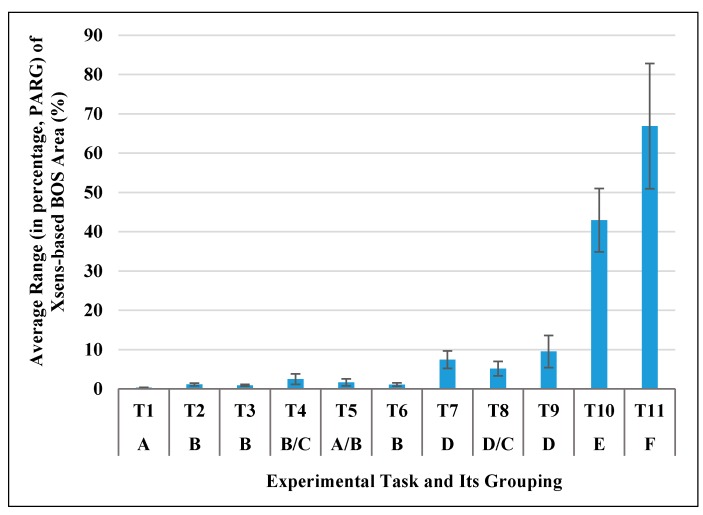
Average range (in percentage, PARG) of Xsens-based BOS area during each experimental task and post-hoc grouping result (Same letters indicate no significant difference).

Figure 8 shows PARG of BOS area. ANOVA test showed that PARG of BOS area was significantly different in the different tasks (*F* (1.324, 11.914) = 161.780, *p* < 0.0005). Post-hoc comparisons showed that the eleven experiment tasks can be categorized into six groups (see A, B, C, D, E and F in Figure 8). The tasks in Groups E and F were dynamic squatting motions, which caused much larger changes in BOS area than the tasks in other groups (Groups A, B, C, D). The tasks in Group D were lower body motions, which caused a relatively larger change in the area than static postures and upper body motions (Groups A, B).

(2) Change in side length

(a) Toe-toe distance and heel-heel distance (medial-lateral direction)

**Figure 9:**
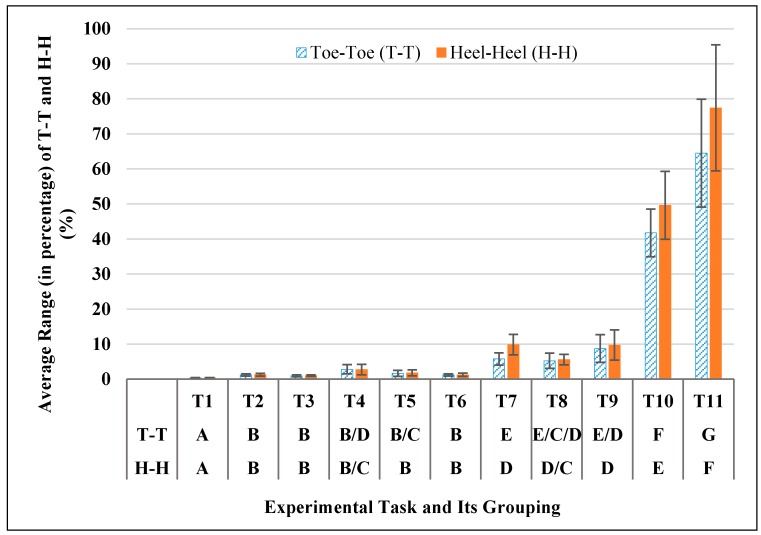
Average range (in percentage, PARG) of Xsens-based side lengths (Toe-Toe, Heel-Heel) during each experimental task and post-hoc grouping result (Same letters indicate no significant difference).

Figure 9 shows the changes in the BOS side lengths. There were significant differences in PARG of T-T and PARG of H-H over different experimental tasks (T-T: *F* (1.333, 11.969) = 167.633, *p* < 0.0005; H-H: *F* (1.364, 112.273) = 166.498, *p* < 0.0005). Post-hoc comparisons showed the eleven experimental tasks can be categorized into seven groups based on PARG of T-T and six groups based on PARG of H-H data (Figure 9). Similar with PARG of BOS area, the tasks in last two groups (T-T: Group F, G; H-H: Group E, F) were dynamic squatting motions and caused a much larger change in BOS side length than the tasks in other groups. And the lower body motions (T-T: Group E, H-H: Group D) caused a relatively larger change in side length than static postures and upper body motions in Groups A and B.

(b) Feet length (anterior-posterior direction)

The changes in foot length during each experimental task was very small with the average PARG of 0.160% (range: 0.035%~0.905%). In addition, there was negligible mean difference between different tasks (all mean differences were within 0.437%).

#### 3.2.2. Change of BOS Position

**Figure 10:**
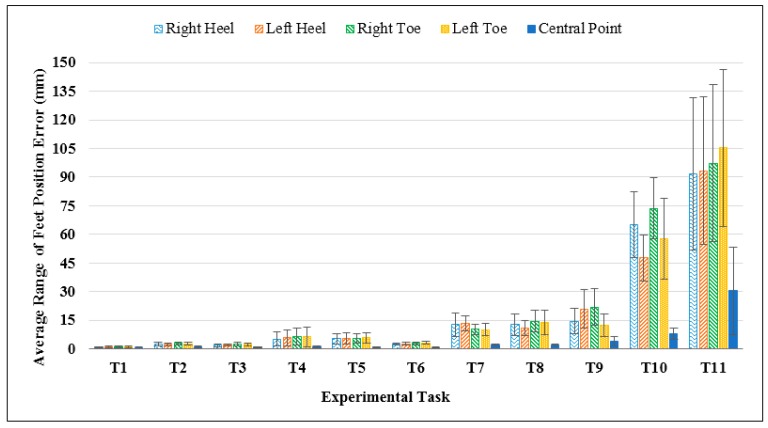
Average range of position errors of Xsens-based BOS in different experimental tasks.

Figure 10 shows the average range (ARG) of BOS position error (PE) in different experimental tasks. Among the eleven tasks, dynamic squatting motions (T10, T11) had the largest effect on the changes of foot positions, and lower body motions took the second place. In all experimental tasks, especially dynamic motions (T4–T11), the position change of central point was much smaller than that of four vertexes (toes and heels), in other words, the position of central point was more stable.

## 4. Discussion

### 4.1. Accuracy of BOS from Footprints 

Footprint-based BOS when both feet were fixed was used as a reference for assessing the accuracy of static BOS from the Xsens system in this study. Since the manual process of making footprints and potential foot movements during experimental tasks could induce considerable errors on BOS measures from footprints, a follow-up experiment was conducted to examine the accuracy of footprint-based BOS, in which an optical camera system was used as the gold reference. Five healthy young males (age 25.0 ± 1.0 years; height 170.4 ± 7.7 cm; weight 71.0 ± 11.0 kg) participated in this experiment. Four reflective markers were attached on the second toes and heels of both feet. OptiTrack V120: Trio (NaturalPoint, Inc. DBA OptiTrack, Corvallis, OR, USA) powered by its matching software Motive (version 1.10.3) was used for data recording at the frequency of 120 HZ. Other experimental settings were the same as in Section 2.2.

**Figure 11:**
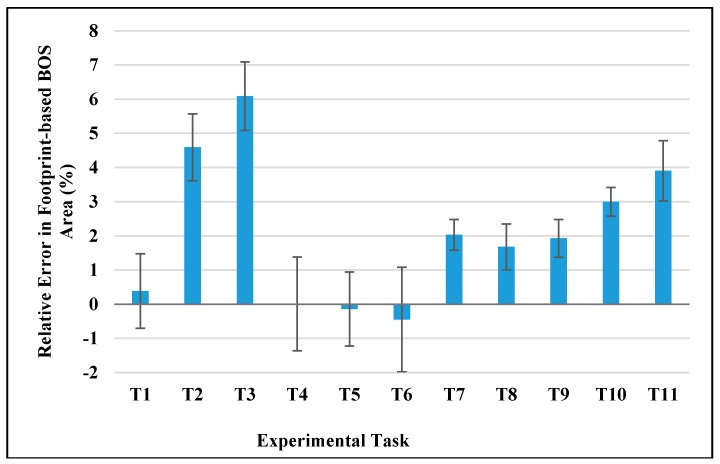
Errors of footprint-based BOS area in different experimental tasks when an optical camera system (OptiTrack V120: Trio) was used as a gold reference.

Figure 11 shows the area difference between the footprint-based BOS and the gold reference. Mean differences for all experimental tasks were within 6.1% (range: 0.01%~6.09%), which were less than 1/5 of the corresponding errors from Xsens-based BOS for all tasks except T10 (see Figure 4). For side lengths of footprint-based BOS, the mean errors were within 2.5% for toe-toe distance (range: 0.03%~2.44%) and 5.8% (range: 0.38%~5.73%) for heel-heel distance. Regarding the position errors of footprint-based BOS, all mean errors were within 14 mm (range: 4.69 mm~13.12 mm) for four vertexes and the central point. These results indicated that errors on static BOS induced from the traditional footprint approach were small and negligible when compared to the identified errors from Xsens MVN system (see Figure 4, Figure 6 and Figure 7).

**Figure 12:**
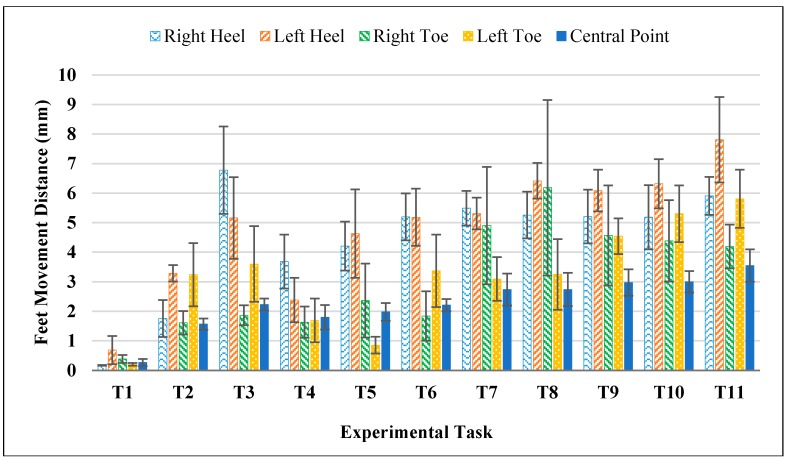
Foot movements in different experimental tasks (an optical camera system and four reflective markers were used for tracking).

The potential foot movements in different experimental tasks were also quantified and the results were shown in Figure 12. Mean foot movements in all experimental tasks were within 8 mm for four vertexes (left toe: 0.20 mm~5.81 mm; right toe: 0.39 mm~6.18 mm; left heel: 0.69 mm~7.81 mm; right heel: 0.17 mm~6.78 mm) and within 4 mm for the central point (0.27 mm~3.54 mm), indicating very small foot movements during the experimental tasks, especially when compared to the identified large position errors from Xsens MVN system (see Figure 7). This result should be reasonable since all the participants were asked to keep their feet still when performing different experimental tasks and the experiment was conducted in a well-controlled manner.

### 4.2. Overall Discussion of the Results

We studied the performance of Xsens MVN BIOMECH in BOS measures (BOS size: area, side lengths; BOS position: vertexes, central point) by comparing with footprint measurements. All the participants were asked to keep their feet still when performing eleven different experimental tasks, therefore, the ideal BOS measures from Xsens MVN should not be different from the reference values from footprints. However, our experimental results showed not only statistically significant, but also practically considerable differences on BOS area (the error ranged from −12.6% to +64.6% depending on the type of experimental tasks). In general, the estimated BOS area from Xsens MVN was smaller than the ground truth from footprints when there was no knee flexion (T1, T4~T9: no squatting); when there was knee flexion (T10, T11, T2 and T3: with squatting), the estimated BOS area from Xsens MVN became larger than the ground truth. There was a clear pattern that Xsens-based BOS area increased monotonically along with the knee flexion angles. Because of that, the accuracy of BOS area firstly increased and then decreased when performing a standing-full squatting task (Figure 5). Almost identical patterns on two side lengths (toe-toe distance, heel-heel distance) can also be observed in Figure 5.

**Figure 13:**
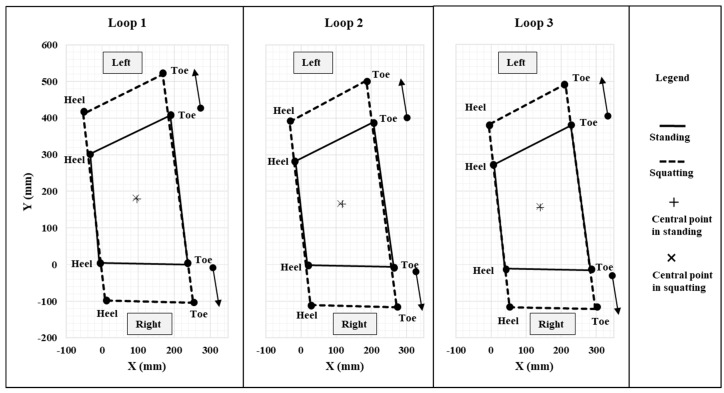
Illustration of the change of Xsens-based BOS in a Standing-Full Squatting motion (Task 11). Note: Central points in standing (+) and in squatting (×) are largely overlapped.

**Figure 14:**
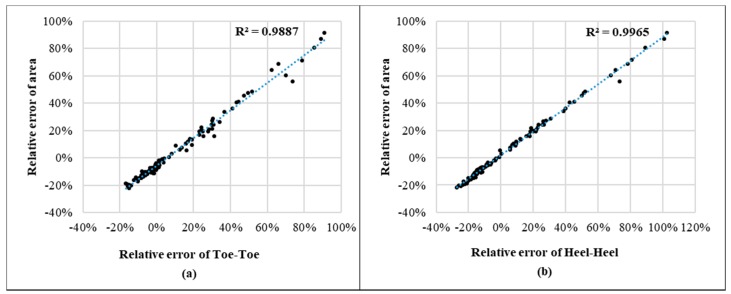
Scatterplots and regression lines of (**a**) Relative error of toe-toe vs. relative error of BOS area, and (**b**) Relative error of heel-heel vs. relative error of BOS area.

Detailed investigations on Xsens data acquired at different experimental tasks revealed interesting patterns on feet movements from Xsens biomechanical model of human along with knee flexion/extension. Figure 13 shows two major features of feet movements from Xsens biomechanical model in a standing to full squatting task: (1) two feet were moving in opposite directions; (2) the magnitude of foot movements was strongly influenced by the knee flexion/extension. The moving directions of both feet were always opposite to each other along the medial-lateral direction, this kind of symmetric movements not only resulted in high variations on positions of four vertexes and thus considerable changes on toe-toe and heel-heel distances, but also maintained a relatively stable position of the central point (Figure 13). A stable position of the central point implies a stable position of center of pressure (COP) if there is no serious unbalanced distribution of ground reaction forces on both feet. Considering the whole body COM position is constantly regulated by the position of COP to maintain the state of human balance [57], the COM position could be relatively stable along with COP, which is consistent with previous studies that reported reasonably good performance in estimating the COM position from Xsens MVN. In addition, the larger knee flexion angle was, the larger foot separation distance (toe-toe, heel-heel distances) became. Linear regression analysis further showed a very high positive correlation between the relative error on BOS area and those on two side lengths along the medial-lateral direction. The relative errors of toe-toe and heel-heel distances explained more than 98% of the total variances of the relative error on BOS area (Figure 14), demonstrating foot movements in the medial-lateral direction was a dominant source for considerable estimation errors on BOS area from Xsens MVN BIOMECH system. This finding should be reasonable since the BOS area is fully determined by toe-toe distance, heel-heel distance, foot lengths (left, right) and their relative orientations. In this study, foot lengths were very accurate (mean error within 0.25%) since they are direct input parameters for Xsens MVN. Accuracy on the orientation estimation of two feet was also high (our data showed that mean errors were within 10° for all experimental tasks) due to sensor-fusion algorithms from inertial sensor based systems including Xsens MVN [11,58,59] and static foot positions during all tasks. Therefore, the estimation error on BOS area was mainly caused by estimation errors on toe-toe and heel-heel distances. Even though the accumulated free drift over time inherent with inertial technologies for estimating positions [10,23,60,61,62] could be another cause of errors on BOS, the error from accumulated free drifts should be negligible when compared with the error from foot movements because of a very short period of time (~20 s) [11,59,60] for each test trial and one new calibration always prior to the test trial.

It is worthwhile to mention that the accuracy of BOS size firstly increased and then decreased in a standing-full squatting task (see Figure 5). We presumed Xsens system has an initial underestimation error on BOS size. To test this assumption, we asked each participant to perform a calibration task (T0) for 20 s, in which he kept the calibration posture (N-Pose) without any movements and data was recorded right after the system calibration. The result showed that Xsens-based BOS size was around 20% (−20.0% in area, −20.0% in Toe-Toe, −19.9% in Heel-Heel) smaller than the real and the left foot deviated more than 40 mm (40.6 mm in heel, 44.4 mm in toe) from its real position, indicating BOS from Xsens MVN system was not accurate even in calibration stage. Moreover, the BOS size was around 10% (−9.7% in area, −4.6% in Toe-Toe, −13.2% in Heel-Heel) smaller than the real in normal standing task (T1). This kind of initial system estimation error was the major reason that why the Xsens-based BOS was always smaller than the real in the experimental tasks (T1, T4~T9) that without squatting-related postures or motions. This error together with wrongly estimated foot movements could explain why the accuracy of BOS size firstly increased then decreased in dynamic squatting motions (T11 and T12).

In addition, the Quadrilateral LA-LC-RC-RA instead of Hexagon LA-LB-LC-RC-RB-RA (see Figure 3b) was used to approximate BOS because it was difficult to mark accurate locations of 5th MTP joints (LB and RB) on the footprints. This approximation underestimated the BOS area since two triangles (ΔLA-LB-LC and ΔRA-RB-RC, areas in red in Figure 3b) were not considered. However, the findings from this study should be still valid due to two reasons: (1) sizes of feet and other body segments from the MVN model are defined by the direct input of anthropometric measures, therefore, they should be accurate and remain stable [26]; (2) foot separation distances (toe-toe, heel-heel) in all experimental tasks were much larger than each foot width, thus the difference between Quadrilateral LA-LC-RC-RA and Hexagon LA-LB-LC-RC-RB-RA should be small. Nevertheless, insole pressure sensors could be used in the future for locating 5th MTP joints so that the hexagon can be generated for more accurate approximation of BOS.

Putting all together, wrongly estimated foot movements along medial-lateral direction from Xsens MVN biomechanical model, mainly caused by knee flexion/extension, and the initial system estimation error on BOS, were two major reasons for the considerable errors and instability of Xsens-based BOS.

### 4.3. Research Implications

As an inertial sensor-based motion capture system, the Xsens MVN BIOMECH has many advantages in terms of portability, ease of use, short setup time and instant data output from its biomechanical model, which makes it being widely used in many fields including biomechanical analysis, sports science, rehabilitation and ergonomics, etc. However, some problems on BOS and foot movements associated with its biomechanical model should not be ignored. It will be good if Xsens MVN BIOMECH developers and researchers can fix this kind of problem by improving their algorithms and biomechanical models. One potential solution to this problem is building a motion database as a knowledge base for later motion reconstruction process [2,3,63,64], specially including some motions with large estimation errors, since our findings showed that motions/postures significantly affect estimation errors of Xsens-based BOS measures. For those who need use this system to obtain BOS or other foot position related measures (such as step width, step length) for different applications, the following measures could be taken to minimize the possible estimation errors based on our findings: (1) if possible, lower body motions, especially both knees’ flexion/extension, should be avoided to the most extent since they can cause large errors and instability in BOS and foot positions; (2) frequent calibration is recommended to reduce the error accumulations over time, especially for reducing errors caused by the accumulation of free drifts; (3) to measure static BOS or foot position in dynamic experimental tasks (like T4–T11 in this study), the following procedure to improve its accuracy and stability is recommended: first ask the subject to place both feet at the target positions and keep erect standing without any movements, then record data for several seconds before performing experimental tasks, the BOS based on the static standing in first several seconds could be used for better accuracy and stability.

### 4.4. Limitations

There were several limitations in the current study. Firstly, even though there were many kinds of body movements in experimental tasks, the BOS in this study was static only since both feet were kept still, future research on dynamic BOS from the Xsens MVN system with an optical camera system as a gold standard should be conducted. Secondly, cautions were taken in this study to minimize possible foot movements when participants performing different tasks, however, feet can’t be fully still and without any movements, which may induce errors on BOS measurements. For example, there were small foot movements for most participants (the average foot movement was within 8 mm for all tasks) when they perform lower body movements, especially in standing with lower body rotation. Thirdly, only healthy young males and a limited sample size were used in this study, thus, the exact estimation errors on BOS measures from Xsens MVN BIOMECH should be used with caution and further studies should be carried out with a larger sample size and other populations for research results verification. Last but not least, even though we have found considerable estimation errors on BOS area and foot separations from Xsens MVN BIOMECH, the underlying causes and possible solutions remain unknown and should be further explored.

## 5. Conclusions

This study investigated the accuracy of static BOS obtained from a widely used inertial sensor based motion capture system-Xsens MVN BIOMECH. Results showed there were considerable errors in estimating BOS area (error ranged from −12.6% to +64.6%, depending on task complexity) from Xsens MVN and a large error in foot separation distance when there was knee flexion. The estimated BOS size (area and side length) from Xsens MVN was smaller than the ground truth from footprint when there was no knee flexion, and larger when there was knee flexion, and it increased monotonically along with the knee flexion angles. Because of that, the accuracy of BOS size firstly increased and then decreased when performing standing to fully squatting motions. Wrongly estimated foot separations, mainly caused by knee flexion, and the initial system estimation error on BOS, were two major reasons for the error and instability of BOS estimation. The findings suggested that caution should be taken when using Xsens MVN BIOMECH to estimate BOS and foot position related measurements, especially for postures/motions with knee flexion.

## Figures and Tables

**Table 1:** Tasks administrated in this study.

Section	No.	Task ^1^	Details of the Task
S1: Static postures	T0 ^2^	Static Calibration Standing	N-Pose calibration posture
	T1	Static Normal Standing	Normal standing posture
	T2	Static Half Squatting	Half squatting posture
	T3	Static Full Squatting	Full squatting posture
S2: Dynamic upper body motions	T4	Standing with Back Flexion and Extension (AP ^3^)	Standing with Back flexion ↔ Back extension
	T5	Standing with Back Lateral Bending (ML ^3^)	Standing with Back lateral bending: left ↔ right
	T6	Standing with Back Rotation (Roll)	Standing with Back rotation: left ↔ right
S3: Dynamic lower body motions (without knee flexion)	T7	Standing with Hip Flexion and Extension (AP)	Standing with Hip flexion ↔ Hip extension
	T8	Standing with Hip Adduction and Abduction (ML)	Standing with Hip movement in ML: left ↔ right
	T9	Standing with Lower Body Rotation (Roll)	Standing with Lower body rotation: left ↔ right
S4: Dynamic squatting (with knee flexion/extension)	T10	Standing—Half Squatting	Standing ↔Half Squatting
	T11	Standing—Full Squatting	Standing ↔ Full Squatting

^1^ Performing above tasks with arms akimbo (except T0, T3, T6, T11) to avoid the interference caused by arm swing, without shoes, and keeping feet still all the time; ^2^ T0 was designed to investigate the initial error of Xsens system (BOS accuracy in calibration posture), in which the data was recorded (20 s) right after performing the calibration; ^3^ AP: anterior-posterior direction; ML: medial-lateral direction.

**Table 2:** Base of Support (BOS) measures from Xsens MVN biomechanical model.

Outcome Measures (Unit)		Definition/Formula
BOS Size	Area (cm^2^)	The area of Quadrilateral LA-LC-RC-RA.^1^
	Toe-Toe (T-T, mm)	The distance between Left 2nd Toe (LA) and Right 2nd Toe (RA).
	Heel-Heel (H-H, mm)	The distance between centers of Left Heel (LC) and Right Heel (RC).
	Feet Length (FL, mm)	Average length of both feet. Foot length is the distance from the heel to the longest toe, when the subject stands with the weight evenly distributed on feet.
BOS Position	Four Vertexes	2D coordinate of 2nd Toes (Left & Right, LA & RA) and Heels (Left & Right, LC & RC) on horizontal plane.
	Central Point (CP)	Average position of four vertexes on horizontal plane: *X*_CP_ = (*X*_LA_ + *X*_LC_+ *X*_RA_ + *X*_RC_)/4; *Y*_CP_= (*Y*_LA_ + *Y*_LC_+ *Y*_RA_ + *Y*_RC_)/4; where (*X*, *Y*) is the coordinate of the 2nd Toe or Heel (Left/Right).

^1^ LA, LC, RA and RC are the points of Left 2nd Toe, Left Heel, Right 2nd Toe and Right Heel of BOS respectively.

**Figure A1:**
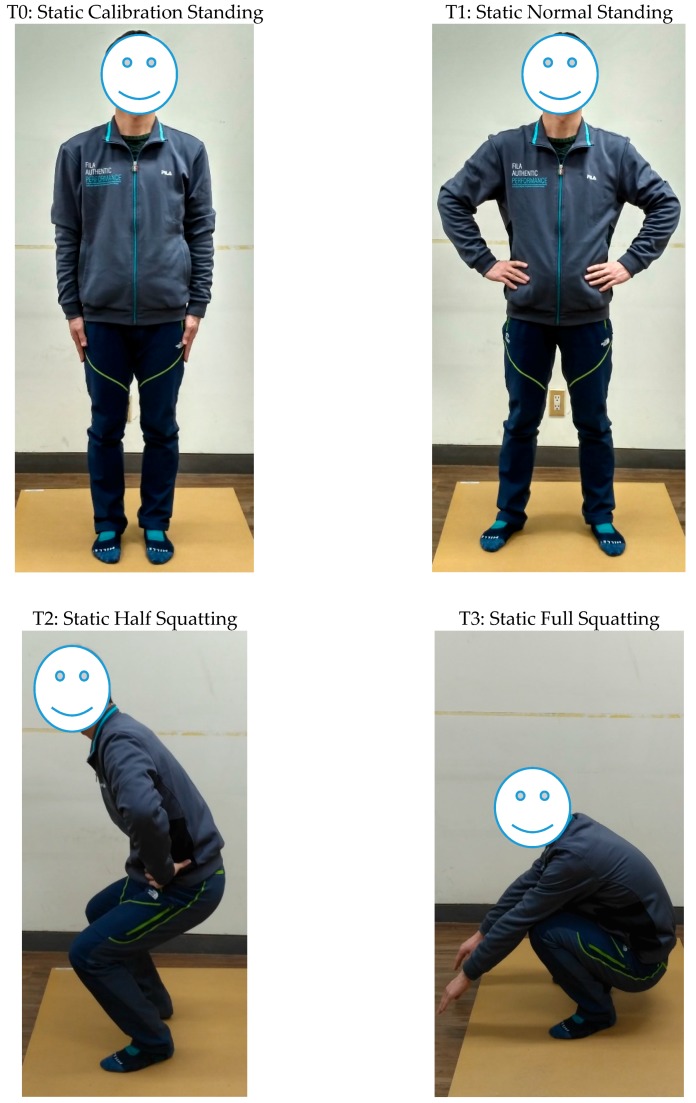

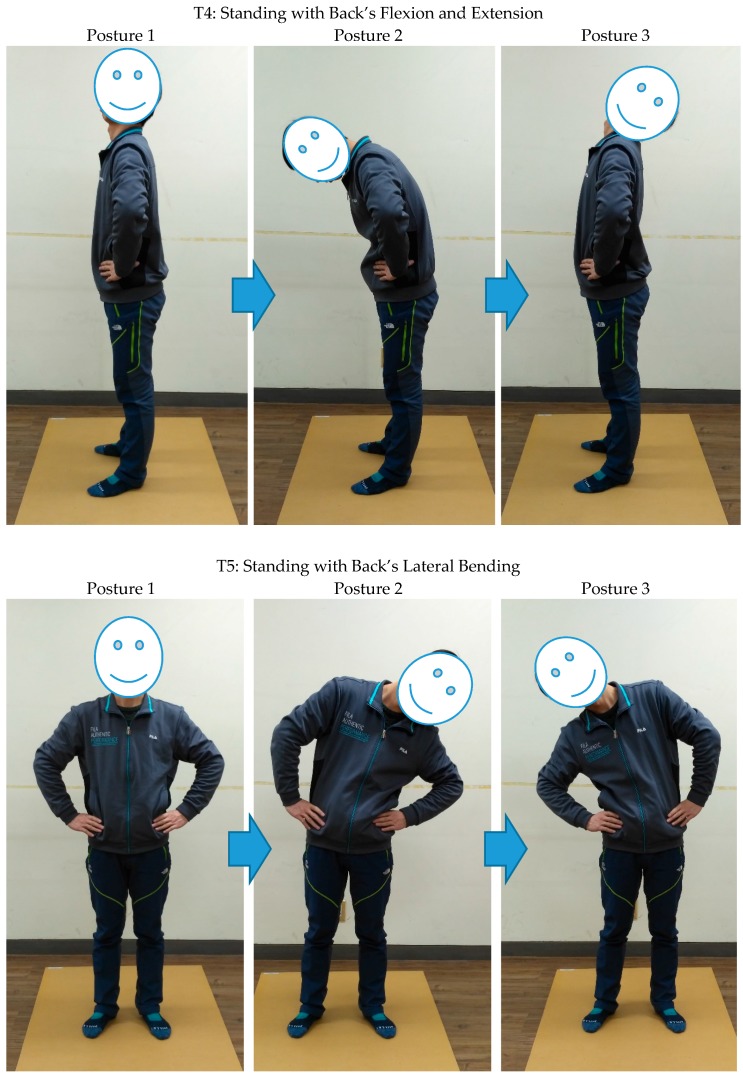

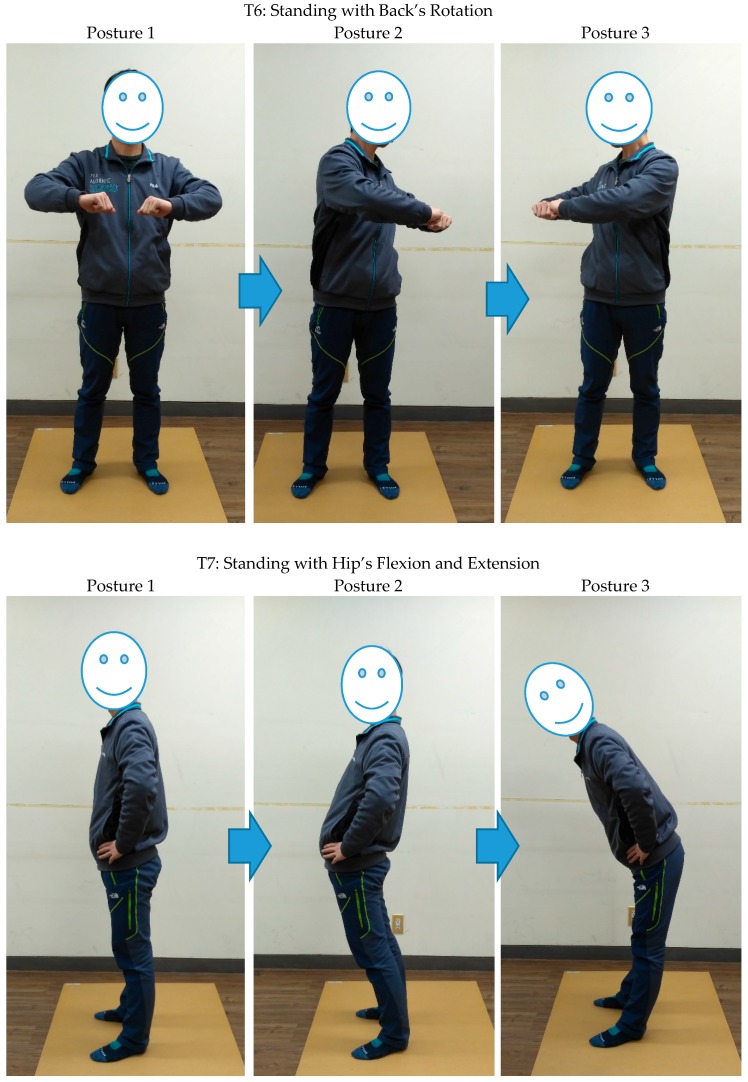

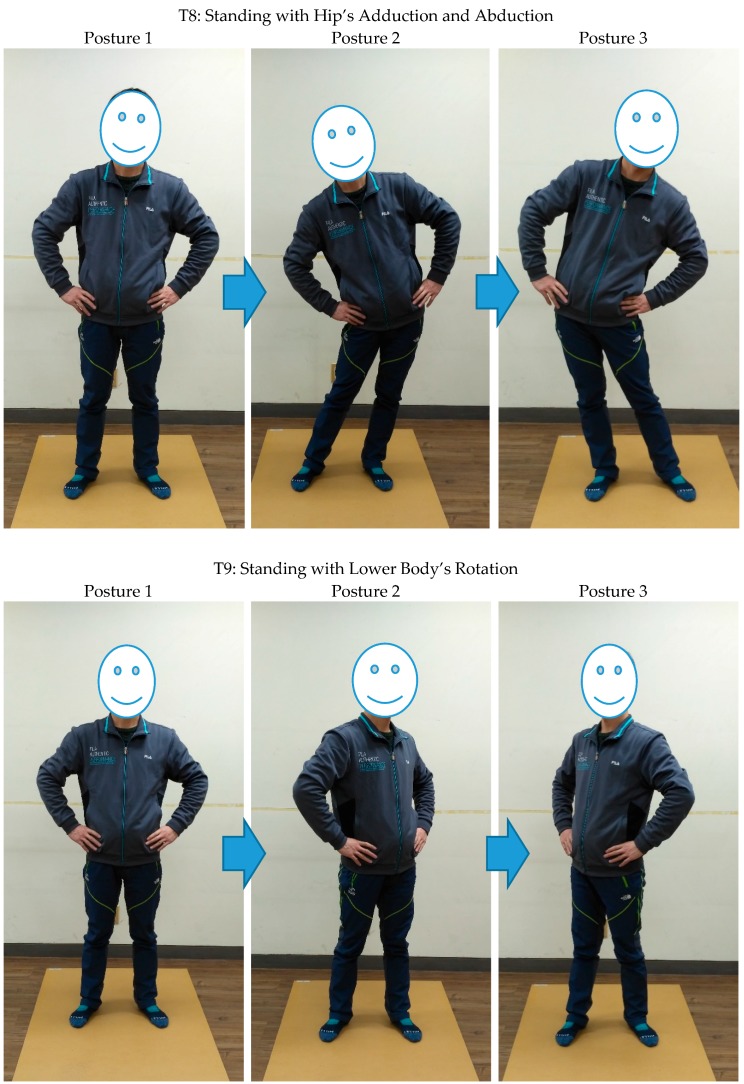

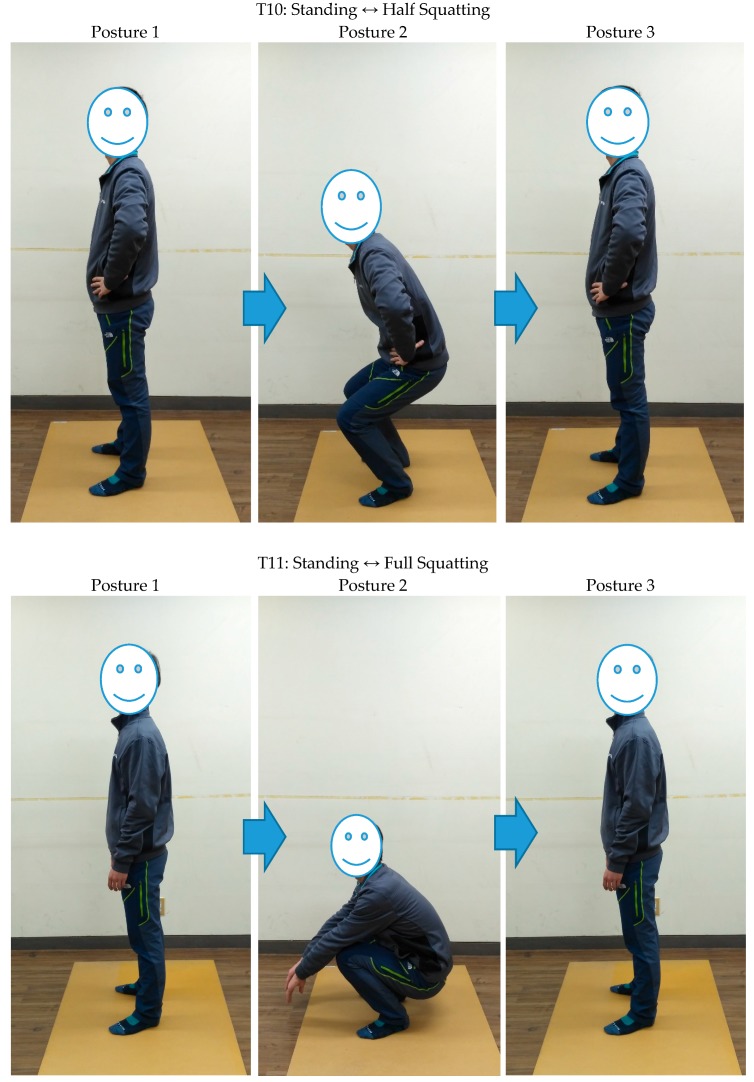
Schematic Representations of the Experimental Tasks.
